# Legume Breeding for the Agroecological Transition of Global Agri-Food Systems: A European Perspective

**DOI:** 10.3389/fpls.2021.782574

**Published:** 2021-11-16

**Authors:** Diego Rubiales, Paolo Annicchiarico, Maria Carlota Vaz Patto, Bernadette Julier

**Affiliations:** ^1^Institute for Sustainable Agriculture, CSIC, Córdoba, Spain; ^2^Research Centre for Animal Production and Aquaculture, CREA, Lodi, Italy; ^3^Instituto de Tecnologia Química e Biológica António Xavier, ITQB-NOVA, Oeiras, Portugal; ^4^Institut National de Recherche Pour l’Agriculture, l’Alimentation et l’Environnement (INRAE), URP3F, Lusignan, France

**Keywords:** legume, breeding, agroecological transitions, agri-food systems, genomic selection

## Abstract

Wider and more profitable legume crop cultivation is an indispensable step for the agroecological transition of global agri-food systems but represents a challenge especially in Europe. Plant breeding is pivotal in this context. Research areas of key interest are represented by innovative phenotypic and genome-based selection procedures for crop yield, tolerance to abiotic and biotic stresses enhanced by the changing climate, intercropping, and emerging crop quality traits. We see outmost priority in the exploration of genomic selection (GS) opportunities and limitations, to ease genetic gains and to limit the costs of multi-trait selection. Reducing the profitability gap of legumes relative to major cereals will not be possible in Europe without public funding devoted to crop improvement research, pre-breeding, and, in various circumstances, public breeding. While most of these activities may profit of significant public-private partnerships, all of them can provide substantial benefits to seed companies. A favorable institutional context may comprise some changes to variety registration tests and procedures.

## The Agroecological Context and the Evolution of Agri-Food Systems

Legume crops are characterized by their high-protein content and their ability to meet their own nitrogen demand by biological nitrogen fixation. Most major legume crops were domesticated at the very onset of agriculture and played a key historical role in agri-food systems as sources of protein complementing carbohydrates provided by cereals, tubers, or roots (Smýkal et al., 2015). Legumes offer specific functional attributes that deliver multiple benefits, including health and nutritional provisions, farmland biodiversity, and improved environmental sustainability (Iannetta et al., 2021). Even when N_2_ fixation was not demonstrated till XIX century (Hellriegel and Wilfarth, 1888), the “reinvigorating” and “manuring” effect of legumes to the soil was already acknowledged by ancient Greek and Roman authors, who recommended their systematic use in crop rotations.

In spite of the known benefits of legume-supported production systems and diets, legume cultivation declined in modern agriculture, particularly in Europe. Grain and forage legumes are grown in only about 1.5 and 1%, respectively, of the European arable area, compared with *circa* 15% in the world. The main reason for that is the spread of and the economic support to intensive cereal-based systems largely relying on the use of nitrogen fertilizers (Watson et al., 2017). Lower economic return from legume crops compared to cereals is frequently claimed by farmers, especially when neglecting the positive effect of a legume on subsequent or companion crops and the opportunity of domestic valorisation. Hence, legumes entered into a vicious circle at all steps of the production and the value chain, resulting in a technological lock-down: the lower production leading to lower use in the value chain, leading in turn to lower investment in agronomy, breeding, farm advisory, and processing, resulting in the increasing marginalization of legume cultivation and use (Magrini et al., 2016). This decline in legume cultivation paired with an increased feed protein demand for livestock and poultry production (Westhoek et al., 2011) has led to about 70% dependence from imported feed protein of Europe, relying essentially on imported soybean to supplement animal diets. This demand, added to the rising feed protein import by China (Gale et al., 2019), contributes to an unsustainable soybean production in South America, leading to wide-scale tropical deforestation (Bager et al., 2020). Conversely, the negative consequences on energy and resource use efficiency, greenhouse gas emissions, nitrogen biogeochemical fluxes, and agricultural biodiversity of not growing legumes locally (Foyer et al., 2016) have largely been overlooked.

The increasing awareness of the non-sustainability of current regional and global agri-food systems is leading to strategic plans aimed to expand legume cropping in the European Union (EC, 2018) and globally (Sivasankar et al., 2016). This is needed to respond to the expected sharp increase in the demand for high-protein food and feedstuff (Pilorgé and Muel, 2016) and the consequent insecurity and high predicted cost of plant protein supply in international market. Another major objective given to legumes is to introduce reactive nitrogen in agriculture. As a consequence, and also because of a high nitrogen demand to grow non-legume crops, agriculture is largely based on the use of nitrogen fertilizers, chemically produced in factories with huge consumption of fossil energy. Meeting this demand of protein production for food and feed and nitrogen input to agriculture is challenging and may require various changes, such as the extension of the cultivation of legumes and a dietary change toward less animal product-based diets (Westhoek et al., 2011; Searchinger et al., 2019; Willett et al., 2019).

The growing social awareness of environmental and health issues has returned legumes to prominence in food systems (McDermott and Wyatt, 2017; Cusworth et al., 2021). We are experiencing a higher demand not only of local vegetable protein for feed but also for food, which, particularly in EU, is associated with an increasing demand for organic and genetically modified free foods and a rise in the adoption of flexitarian, vegetarian, and vegan diets (EC, 2018). Legumes are in fact a key component of the Mediterranean diet, which is extending to other areas and is driving a culinary revival of local produce and home cooking. At the same time, legumes are entering rapidly into the processed food business (Cusworth et al., 2021). Legume-derived protein is less expensive relative to meat, egg, and dairy proteins and is amenable to a wide variety of processing applications whose production sector has a two digits’ annual growth (EC, 2018).

The positive impact of legume-rich grasslands on ruminant feeding and ecosystem services is increasingly acknowledged (Martin et al., 2020). Perennial legumes, compared with annual grain legumes, offer the additional advantage of maximizing the production of proteins per unit area by a factor of 2–4. The relative inefficiency of extensive livestock systems in terms of greenhouse gas emissions ought to be weighed against the highly positive effects of these systems on landscape preservation, animal and crop diversity, resource use recycling, and production of typical or organically produced food with high added value (Swagemakers et al., 2017), suggesting to rather safeguard and improve the efficiency of these systems by greater reliance on legume-rich perennial grasslands.

As a result, legume cultivation may speedily recover, and predictions are for its further increase. This should be paired with technical solutions to support this growth, making legumes competitive for land at produce prices. This can only be achieved through an integrative approach leading to the adjustment of cropping practices and the breeding of more adapted, attractive, and productive cultivars able to address producers and consumers’ needs.

## Breeding for Increasing Crop and Cropping Systems Diversity

Increasing legume cropping requires a breeding investment on a large number of species (Table 1) adapted to the diverse agro-climatic conditions and product uses. A broad classification of major species can be made depending on their main use (food, feed, or cover crop), crop cycle (annual or perennial), and breeding system (allogamous or autogamous). Species grown in temperate regions mostly originated from the Fertile Crescent and first expanded in the Mediterranean region (with the exception of soybean, of Chinese origin, and common bean and one lupin species, of American origin). Most of them are relatively well adapted to northern latitudes, because of cold tolerance and/or cultivation as summer crops.

**Table 1:** Major legume crops ranked by the number of cultivars registered in the EU as an indication of breeding efforts devoted to them.

Major crop species		No. of registered cultivars		No. of accessions in geneBanks		Genomic resources available (reference genome sequence or a recent review on the subject)
Scientific name	Common name	EU^1^	Globally^1^	European^2^	Globally^3^	
*Phaseolus vulgaris*	Common bean	3.728	5.956	49.662	136.167	Assefa et al., 2019
*Pisum sativum*	Pea	3.377	5.959	35.425	55.732	Kreplak et al., 2019; Pandey et al., 2021
*Glycine max*	Soybean	1.947	15.430	16.108	83.592	Chu et al., 2021
*Medicago sativa*	Lucerne	1.270	3.680	8.485	18.353	Chen et al., 2020
*Vicia faba*	Faba bean	830	1.398	13.229	26.044	Khazaei et al., 2021
*Trifolium pratense*	Red clover	739	1.485	10.507	11.548	De Vega et al., 2015
*Trifolium repens*	White clover	430	1.054	4.960	9.600	Griffiths et al., 2019
*Vicia sativa*	Common vetch	312	665	8.220	13.189	Zhu et al., 2019
*Phaseolus coccineus*	Runner bean	191	273	2.435	5.226	Xanthopoulou et al., 2019
*Lupinus angustifolius*	Narrow-leafed lupin	177	357	3.110	5.096	Kamphuis et al., 2021
*Cicer arietinum*	Chickpea	120	213	12.015	63.612	Roorkiwal et al., 2020
*Trifolium incarnatum*	Crimson clover	118	178	219	401	No info
*Trifolium alexandrinum*	Berseem clover	103	184	243	648	No info
*Lotus corniculatus*	Lotus	98	229	2.006	3.628	Mun et al., 2016
*Lupinus albus*	White lupin	96	174	4.043	5.490	Kamphuis et al., 2021
*Onobrychis viciifolia*	Sainfoin	55	87	690	1.179	Shen et al., 2019
*Trifolium resupinatum*	Persian clover	52	97	324	1.889	No info
*Trifolium subterraneum*	Subterranean clover	36	110	5.846	16.124	Hirakawa et al., 2016
*Lupinus luteus*	Yellow lupin	48	85	3.301	3.781	Iqbal et al., 2020
*Lens culinaris*	Lentil	33	250	9.113	30.846	Guerra-García et al., 2021
*Arachis hypogaea*	Groundnut	29	576	3.320	32.933	Bertioli et al., 2019
*Hedysarum coronarium*	Zulla	22	35	126	412	No info
*Lathyrus cicera*	Red pea	17	27	700	1.183	Santos et al., 2018
*Trigonella foenum-graecum*	Fenugreek	17	36	379	1.212	No info
*Medicago lupulina*	Black medic	11	19	594	1.489	No info
*Ornithopus sativus*	Bird’s foot	10	31	230	708	No info
*Vicia ervilia*	Ervil	8	11	1.245	2.162	No info
*Vicia narbonensis*	Narbon bean	7	22	593	1.247	No info
*Vigna unguiculata*	Cowpea	6	81	4.144	40.384	Lonardi et al., 2019
*Lathyrus sativus*	Grass pea	6	40	3.671	7.663	Emmrich et al., 2020
*Medicago polymorpha*	Burr medic	6	23	1.012	9.144	Cui et al., 2021
*Ornithopus compressus*	Yellow bird’s foot	4	15	906	3.297	No info
*Medicago truncatula*	Barrel medic	2	19	2.253	9.309	Pecrix et al., 2018
*Vigna radiata*	Mungbean	2	49	1.086	15.944	Ha et al., 2021

1 CPVO, 2021 (http://cpvoextranet.cpvo.europa.eu).

2 EURISCO, 2021 (https://eurisco.ipk-gatersleben.de/).

3 GENESYS, 2021 (https://www.genesys-pgr.org/).

Breeding relies on genetic diversity, of which the maintenance in Genetic Resources Centers is crucial. At the European and world levels, wild and cultivated (landraces, old varieties) accessions of legume species are stored, multiplied, and shared (Table 1). Better knowledge on these accessions (phenotypic and genotypic data) is useful to make use of these genetic resources in breeding programs. The selection of varieties, while aiming to maximize breeding progress, tends to progressively reduce the genetic diversity used in agriculture. Breeding may also be challenged by insufficient diversity for specific emerging traits. This can be remedied by a continuing infusion of genetic diversity coming from old varieties, landraces, ecotypes, or wild populations. However, legume breeding programs are relatively small, often forcing breeders to fulfill short-term breeding goals by using elite germplasm rather than “exotic” germplasm, which may require lengthy pre-breeding (Dwivedi et al., 2016; Coyne et al., 2020; Pratap et al., 2021). This emphasizes the importance of pre-breeding programs carried out by public institutions that exploit landraces and wild relatives and make the resulting germplasm globally available.

There is increasing interest by some farmers of the organic sector for greater cultivation of seeds of genetically heterogeneous material of inbred crops rather than pure line cultivars, because of the advantages in terms of crop resilience and yield stability that genetic diversity is expected to provide. This conflicts with the genetic uniformity required for cultivar registration. Recently, the EU has allowed to market “Organic Heterogeneous Materials,” not fulfilling the cultivar requirements as regards uniformity under certain conditions (EU, 2018, 2021). These might include farmers’ selection from populations or landraces, dynamic populations, and composite cross populations, devised to get progressively adapted to specific farm conditions under which they evolve, as a kind of modern farm ecotypes (Costanzo and Bickler, 2019). The strategy to grow heterogeneous material conflicts with the breeding theory that aims to produce the best genotype and suggests that micro-niches are exploited by different genotypes. The situations (stress, spatial heterogeneity) in which a certain extent of diversity is favorable to yield and yield stability are an important area of future research. Multi-environment comparisons of line mixtures vs. their best component pure lines indicated the possibility to grow mixtures with higher yield stability and just a modest yield penalty for soybean (Carneiro et al., 2019), and the presence of an optimal level of genetic diversity that allows to increase the crop yield stability without a penalty on crop yield for the inbred forage subterranean clover (Pecetti et al., 2020).

## Breeding for Emerging New Traits and Diversified Target Uses and Environments

Breeding needs to identify and exploit genetic variation for relevant traits supported by innovative phenotyping tools and molecular markers-based procedures to develop “smart” cultivars that yield more with low inputs. Key traits for grain or forage legume breeding were summarized in previous reviews (e.g., Annicchiarico et al., 2015a; Araújo et al., 2015; Duc et al., 2015; Rubiales et al., 2015; Vaz Patto et al., 2015). Hereafter, we focus on emerging traits as suggested by the changing climate and the needed agroecological transition.

The agroecological transition requires not only greater legume crop cultivation in rotations but also greater exploitation of legume-based intercrops, which, in modern agriculture, has been widespread only for some perennial legumes, especially white clover. The association of annual legumes with cereals, or that of perennial legumes with forage grasses, can exploit plant functional diversity to raise crop yields, yield stability, and/or crop quality, while simultaneously enhancing ecosystem services and reducing adverse environmental impacts (Martin-Guay et al., 2018). Breeding for intercropping may be challenging for legume breeders, requiring the identification of trait mean and variance that are responsible for the survival and the production of a target species grown with one or several companion species (Maamouri et al., 2017). Specific breeding for intercropping is supported by several studies on perennial legumes reviewed in Annicchiarico et al. (2019a), as well as by recent findings for an annual legume, such as pea (Annicchiarico et al., 2021).

Greater adaptation to low input conditions will be a leading priority in legume breeding, particularly for organic systems that are on the rise in EU with a mandate to reach 25% of the EU cropping area by 2030. Improved symbiosis and nitrogen use efficiency are therefore becoming compulsory (Sreeharsha et al., 2021). Phosphorous (P) use efficiency is also gaining importance as we are facing an alarming decline in the availability of natural sources of P for soil amendments. Efficiency in S, K, and Zn uptake can be a priority for certain soil types, especially where interactions with P- and N-use efficiency affect the final nutritional quality of the grain (Blair, 2013).

The global change and increasing instability of the climate pose additional challenges to breeders (Andrews and Hodge, 2010). It emphasizes a need for greater tolerance to major biotic (Rubiales et al., 2015) and abiotic stresses (Araújo et al., 2015). Tolerance to drought will have even greater importance due to lower rainfall as well as lower available irrigation water in many regions (e.g., Polade et al., 2017). Tolerance to low winter temperatures of autumn-sown crops remains a key target in many regions despite the climate change, because occasional low temperatures on poorly hardened plants issued by a mild-winter period can be highly damaging (Araújo et al., 2015), but also because of the requested expansion of legume cultivation toward northern regions (Ergon et al., 2018) or anticipated sowing aimed to crop escape from summer drought. Heat waves at flowering and grain filling stage are an increasing threat, with heat tolerance becoming a priority not only in hot regions but also in spring sowings in temperate regions and even in winter sowings in Mediterranean Basin at low altitudes (Rubiales et al., 2021). Pest and diseases are becoming more and more critical in the predicted scenario of sharp decrease in pesticide uses during cultivation and storage. Also, global changes will affect the relative importance of pests and diseases impacting their geographic distribution (especially northward) and frequency of outbreaks by affecting their overwintering survival and ability to develop more generations (Skendžić et al., 2021). For instance, pod borer insects (Sharma et al., 2020) and the parasitic weed broomrape (Rubiales, 2020) are expected to extend northward.

Climate change-related stresses will have a negative impact not only on yield but also on nutrient quality of grain legumes (Scheelbeek et al., 2018) with a clear nutritional quality ranking change for legume varieties under, for example, heat stress conditions (Mecha et al., 2021). Conversely, global warming should extend the period of production of perennial grasses, enabling an increase in annual forage yield in spring and autumn, although with risks on summer survival in some regions or years (Durand et al., 2010). In forage legumes, drought has a combined effect on protein content: The reduction in biomass is associated to protein content increase but drought may limit nitrogen fixation (Lemaire and Allirand, 1993).

The predicted increase of legume-based food consumption due to the rise of plant-based and flexitarian diets and the planned EU reduction of meat consumption will widen the economic opportunities for domestic cultivation of grain legumes, since food use provides greater added value than feed use. Until recently, food legume breeders’ efforts have focused mainly on improving protein yield and reducing “undesirable” compounds contents (such as raffinose family oligosaccharides in lentils, phytates in pea, vicine-convicine in faba beans, and ODAP in grass pea), while largely neglecting sensory or processing important traits (Duc et al., 2015; Vaz Patto et al., 2015). The insufficient attention paid to the integrated approach needed for improving food legume quality resulted in a lack of innovation and low attractiveness of legume food products that, together with the emergence of novel food habits, resulted in a reduction in consumption (Vaz Patto et al., 2015). To reverse this trend, consumer’s preference should be a major driver in food legume breeding. In the more sustainable (less energy needed during processing, with reduced wastes), minimal or mild processed legume-based foods (whole seed or flour consumption), besides appearance, the basic taste qualities (sweet, sour, bitter, salty, and umami), and certain texture properties are of most importance (Roland et al., 2017). Moreover, taste, aroma, and appearance are significantly associated with specific metabolites content in foods (Liem and Russell, 2019; Mecha et al., 2022) that might also influence their final nutritional and health benefits, which are aspects with increasing importance on consumers’ choices. An example is the antioxidant phenolic compounds content in food legumes (Mecha et al., 2019). Sensory analysis cannot be attained unless pulses are cooked. In this way, besides the conventional cooking time determination, expedite sensory methodology combining the choice of product’ attributes with their intensity scale classification are needed to overcome cost and time constraints of conventional approaches (Ares et al., 2014). When considering highly processed legume-based foods, involving isolated macromolecular fractions of the flours (isolates and concentrates), breeding objectives might focus on increasing the contents and isolation yields of the most valuable nutrient. Examples are protein, fiber, and starch concentrates or isolates (Vaz Patto et al., 2015) used on the development of nutraceuticals, functional foods, ultra-processed foods made of recombined ingredients (such as meat and dairy products replacers), and nutritional supplements. Although not as sustainable as the minimal or mild processed legume-based food system, this is a rapidly growing market, attracting a new and mainstream audience (Cusworth et al., 2021). In any of the cases (minimal or highly processed legume-based food products), an increased food use will require collaborative developments between legumes breeders and the food industry, exploiting in the most efficient way the existing diversity in quality traits both within and between grain legume species.

For forage crops, useful new quality traits may include increased Omega-3 polyunsaturated fatty acids, vaccenic acid, and conjugated linoleic acid, with reduced levels of omega-6 fatty acids and palmitic acid in milk (Alothman et al., 2019). Considering the ruminants as consumers, breeding for increased content in condensed tannins is important to improve protein valorization in the digestive tract, reduce N release in the environment, and decrease the impact of parasitic nematodes (Mueller-Harvey et al., 2019).

Farmer’s acceptability of cultivars cannot be considered a new trait by itself, but farmer-participatory selection is gaining increasing attention especially for organic farming. There is a paucity of formal assessments of its value for countries with developed agriculture, but its application to a large-scale pea breeding program in Italy produced greater yield gains than ordinary breeder’s selection (Annicchiarico et al., 2019c).

Adaptation for use as a service (alias cover) crop is a further new trait of interest for some legume species in the European agriculture. Two main situations are encountered as: Either a crop (or a crop mixture) is grown in summer just after the last harvest until the new sowing (in autumn, end of winter, or spring) to catch residual nitrogen, control weeds and pests, avoid soil erosion, and improve the soil carbon content, or a crop is grown together with a cash-crop as a service-provider only. Annual legumes for these uses should possess rapid summer establishment and autumn growth (e.g., faba bean and crimson clover). Perennial legumes may offer a living mulch for grain crops over several years (e.g., white clover and prostrate-type lucerne). The breeding of these crops is challenged by greater interest of minor species or plant types and the use of test conditions and target traits that are completely different from those currently used as grain or forage legumes (e.g., evaluation of soil coverage, summer establishment, early vegetative growth, nitrogen content in the biomass, capacity to be destroyed by winter frost, and low competition with the companion grain crop). In addition, their seed cost ought to be very low.

## Novel Techniques to Enhance the Selection Efficiency and Ease the Complexity of Selection

As outlined in the earlier sections, legume breeders have to cope with ever-increasing quantitative target traits and the need to reduce the profitability gap with major cereals by drastic yield improvements, in the presence of modest budgets. Field phenotyping remains a bottleneck for crop genetic improvement. Legume improvement can today be supported by the integration of modern genomics approaches, high-throughput phenomics, and simulation modeling (Araus et al., 2018; Varshney et al., 2018). The challenge for wider adoption by breeders is the cost per sample, which is today lower for genotyping than for field-based phenotyping and for high-through phenotyping platforms. The emergence of novel non-destructive root/aerial phenotyping methods can support selection for improved symbiosis as well as other agronomic traits. Affordable low-cost phenotyping tools are being developed, including unmanned aerial vehicles and sensors mounted on “phenomobiles” and can be particularly valuable to decrease the cost of field evaluations (Cazenave et al., 2019).

The use of markers in breeding programs got impulse from the development of high-throughput genotyping techniques, such as genotyping-by-sequencing (GBS), and large SNP array tools. Besides the QTL identified in restricted parental crosses that are hardly used in breeding programs, genome-wide association studies (GWAS) provided markers that explain trait variation in a chosen population. These QTL explain a portion of genetic variance, useful to track major alleles. Most agronomically important traits are polygenic and, as such, can greatly profit of genomic selection (GS), which enables small-effect loci to be incorporated into prediction equations. An interesting option would be to combine in a single model both QTL and all the markers covering the genome, through procedures that may also allow to define a subset of highly explanatory SNPs for inclusion in less expensive selection tools (Li et al., 2018).

A key question for legume breeders is whether GS may have greater selection efficiency than phenotypic selection for crop yield improvement. Pioneer studies highlighted greater predicted yield gain per unit time or unit cost for alfalfa (Annicchiarico et al., 2015b), soybean (Matei et al., 2018), and pea (Annicchiarico et al., 2019b). In legumes, GS displayed convenient predictive ability also for key grain quality (Stewart-Brown et al., 2019) or forage quality traits (Biazzi et al., 2017; Pégard et al., 2021) and emerging complex traits, such as drought tolerance (Li et al., 2019; Annicchiarico et al., 2020), performance in intercropping (Annicchiarico et al., 2021), and tolerance to some biotic stresses (Carpenter et al., 2018). However, research work is crucially needed to fully assess the potential of GS for different legume species and target traits, explore the transferability of its models to different breeding populations, and optimize its adoption within the breeding schemes. GS has high potential importance for breeding programs not only to enhance their selection efficiency but also to ease the cost and complexity of breeding for several target traits, once prediction equations are available. The number of evaluated genotypes for a fixed trait selection fraction increases exponentially as a function of the number of target traits, quickly reaching genotype numbers whose evaluation cost is beyond reach for phenotypically evaluated traits. GS selection costs (which depends on the genotyping cost) are nearly zero from the second target trait onwards.

Variant detection (or allele mining) is also a way to identify genes involved in trait variation and alleles conferring a positive effect on a trait (Kumar et al., 2010). For autogamous species in which accessions are mostly pure lines, the detection of variants requires to investigate large panels of genetic resources. For allogamous species, variants, including lethal or sublethal ones at an evolutionary meaning, may be present at a heterozygous status. Such variants could be interesting in breeding, as exemplified for genes involved in lignin content in lucerne (Gréard et al., 2018). Prediction models can be useful also for cost-efficient mining of key traits in large germplasm collections (Jarquín et al., 2016). More generally, markers can bring more progress compared to phenotypic selection in heterozygous and more over tetraploid species, such as lucerne, lotus, and some clovers, because the recessive alleles conferring a positive effect can be selected (Julier et al., 2003).

## Institutional and Research Policy Aspects

Wider and more profitable legume crop cultivation is an indispensable step for the agroecological transition of global agri-food systems but represents a challenge especially in Europe. Plant breeding is pivotal in this context. Variety breeding is reputed to be a fruitful business, enabling economic activity through seed marketing and genetic progress. However, when the surfaces are low, the return of the breeding activity is not sufficient to support strong breeding programs. Taking the number of registered legume cultivars (CPVO, 2021; Table 1) as an indication of the breeding efforts devoted to each species, it seems that the EU seed business is mostly active on common bean, pea, soybean, and faba bean among the grain legumes, and on lucerne, red clover and white clover among the forages. Even for the so-called major legume species (pea, faba bean, and lucerne), the breeding programs are less ambitious than on other non-legume crops, such as maize, sunflower, and oilseed rape.

Reducing the profitability gap of legumes relative to major cereals will not be possible in Europe without public funding devoted to crop improvement research and pre-breeding activities and, in various circumstances and especially for minor crops, public breeding. While most of these activities may profit of significant public-private partnerships, all of them can provide substantial benefits to seed companies (in the form of transferred breeding techniques, genetic resources, or varieties to be marketed). A favorable institutional context ought to comprise variety registration procedures and marketing regulations able to value innovative variety traits and to reverse, under specific circumstances, the trend toward the cultivation of genetically uniform varieties. In Europe, only France has a levy system on grain legume production (pea, faba bean, and lupin grown from certified or farmer’ seed) when collected by a cooperative and allocated to a fund that supports plant breeder’s projects. This tax, known as “Cotisation Volontaire Obligatoire” (Mandatory Voluntary Contribution), is not applied (yet) to other grain legumes and does not apply to forage legumes, which are mostly not collected but used directly on farm to feed cattle. Similar systems apply in Canada and Australia, for instance, with returns to public breeding programs, mainly held by Universities, leading to continuous funder-led breeding improvements. Europe’s policy makers should explore this further, facilitating public-private breeding interaction for the good of all. The lack of sufficient economic returns also explains the fact that public breeding programs still are the main contributors to legume crop improvement in most European countries. In any case, the support of public research institutions is crucial for the development of pre-breeding or breeding activities and innovative selection procedures (such as GS), as a form of research policy-supported public-private partnership.

At the moment, Genetically Modified Organisms (GMO) in Europe are submitted to complex regulation although are they are used for scientific research. Moreover, plants derived from genome editing enter into GMO category at the moment (Court of Justice of the European Union, 2018). Changes in current regulations and public perceptions are needed before these techniques can be widely adopted by breeders in Europe. Also variety registration procedures may require improvement in various respects, to verify and enforce the genetic progress performed by breeding programs. Important areas needing improvement include the assessment and adequate quantification of the importance of emerging traits related to crop quality, stress tolerance, or suitability for intercropping. A pan-European variety testing network, organized at the level of agrizones with a shared contribution of registration offices, could be a way to improve variety evaluation, increase the number of traits and conditions under study with a controlled budget (Gilliland et al., 2020). In addition, molecular marker-based procedures for variety distinctness, especially for species bred as synthetic varieties, such as lucerne, could help to solve the situation in which a genetic progress is proved but variety distinction, based on morphological traits, fails (Julier et al., 2018; Gilliland et al., 2020).

## Author Contributions

All authors listed have made a substantial, direct and intellectual contribution to the work, and approved it for publication.

## Funding

This work was supported by Spanish AEI project PID2020-11468RB-100, Italian MIPAAF project GENLEG, EU-H2020-EUCLEG project 727312, and Portuguese FCT project UID/04551/2020.

## Conflict of Interest

The authors declare that the research was conducted in the absence of any commercial or financial relationships that could be construed as a potential conflict of interest.

## Publisher’s Note

All claims expressed in this article are solely those of the authors and do not necessarily represent those of their affiliated organizations, or those of the publisher, the editors and the reviewers. Any product that may be evaluated in this article, or claim that may be made by its manufacturer, is not guaranteed or endorsed by the publisher.
